# HMCDA: a novel method based on the heterogeneous graph neural network and metapath for circRNA-disease associations prediction

**DOI:** 10.1186/s12859-023-05441-7

**Published:** 2023-09-11

**Authors:** Shiyang Liang, Siwei Liu, Junliang Song, Qiang Lin, Shihong Zhao, Shuaixin Li, Jiahui Li, Shangsong Liang, Jingjie Wang

**Affiliations:** 1 Department of Gastroenterology, Tangdu Hospital, Air Force Medical University, Xinsi Road, Xi’an, China; 2 Department of Machine Learning, Mohamed bin Zayed University of Artificial Intelligence, Abu Dhabi, United Arab Emirates; 3 Department of Respiratory Medicine, Tangdu Hospital, Air Force Medical University, Xinsi Road, Xi’an, China; 4 Department of Internal Medicine, The No. 944 Hospital of Joint Logistic Support Force of PLA, Xiongguan Road, Jiuquan, China

**Keywords:** Heterogeneous graph neural network, Metapath, CircRNA, Disease

## Abstract

Circular RNA (CircRNA) is a type of non-coding RNAs in which both ends are covalently linked. Researchers have demonstrated that many circRNAs can act as biomarkers of diseases. However, traditional experimental methods for circRNA-disease associations identification are labor-intensive. In this work, we propose a novel method based on the heterogeneous graph neural network and metapaths for circRNA-disease associations prediction termed as HMCDA. First, a heterogeneous graph consisting of circRNA-disease associations, circRNA-miRNA associations, miRNA-disease associations and disease-disease associations are constructed. Then, six metapaths are defined and generated according to the biomedical pathways. Afterwards, the entity content transformation, intra-metapath and inter-metapath aggregation are implemented to learn the embeddings of circRNA and disease entities. Finally, the learned embeddings are used to predict novel circRNA-disase associations. In particular, the result of extensive experiments demonstrates that HMCDA outperforms four state-of-the-art models in fivefold cross validation. In addition, our case study indicates that HMCDA has the ability to identify novel circRNA-disease associations.

**Supplementary Information:**

The online version contains supplementary material available at 10.1186/s12859-023-05441-7.

## Introduction

Circular RNA (circRNA) is a class of non-coding RNA which neither have a 5’-terminal hat nor a 3’-terminal poly (A) tail. In particular, circRNA is formed by the ring structure with covalent bonds. Since the first circRNA was discovered in 1971, researchers have identified more than 183,000 circRNAs from human transcriptome [1–4]. Recently, researchers have found that circRNAs can serve as biomarkers and targets of treatment for many diseases. For example, Zang et al. [5] found that circRNA EIF4G3 could suppress gastric cancer progression through the inhibition of $\beta$-catenin. Young et al. [6] found that circ_0023984 could promotes the progression of esophageal squamous cell carcinoma by sponging miR-134-5p. Therefore, it is critical to identify circRNA-disease associations (CDAs). Verification of the relationship between circRNAs and diseases usually require a large number of experiments such as reverse transcription-PCR (RT-PCR) [7, 8], northern blotting [9, 10], nucleus/cytoplasm fractionation [11, 12]. The prediction results of high accuracy can provide the correct direction for the basic experiments and reduce the cost of the experiment.

Currently, there are a large number of associations between circRNAs and diseases that have been verified by experiments, and have been carefully collected as formatted data by professionals. For example, Lei et al. [13] have constructed the CircR2Disease database by collecting the CDAs verified by experiments. As of the latest version [13], the database includes 4201 associations between 3077 circRNAs and 312 diseases.

Thanks to the rapid development of computational technology and the collection of a large number of experimental data, researchers have proposed many methods to predict new CDAs [14–19]. The previous prediction methods can be divided into three categories: methods based on similarity, methods based on matrix decomposition, and methods based on graph neural networks (GNNs) [20–23].

The underlying intuition of the methods based on similarity is that similar circRNAs are associated with similar diseases. Based on this intuition, researchers calculated the similarities between circRNAs and the similarities between diseases using a variety of data sources. After that, these similarity data and the verified relationships are used to predict novel relationships. For example, Wang et al. [15] constructed the disease similarities by integrating the disease semantic similarity, disease Jaccard similarity and Gaussian kernel similarity. Then the circRNA similarities were constructed by integrating the Jaccard similarity of circRNAs and the Gaussian kernel similarity of circRNAs. Based on these similarities, they proposed a model named IMS-CDA (Prediction of CDAs From the Integration of Multisource Similarity Information With Deep Stacked Autoencoder Model) to predict the associations between circRNAs and diseases.

The second type is method based on matrix decomposition. Li et al. [24] proposed a method based on Speedup Inductive Matrix Completion (SIMCCDA) to predict the potential relationship between circRNAs and diseases. In particular, the proposed SIMCCDA model treats the circRNA-disease matrix as an observed matrix with missing values; hence the task is to predict those missing values by decomposing this observed matrix as two lower dimensional matrices.

Meanwhile, the third type is a method based on GNNs. With the development of GNNs, many researchers use GNNs to predict CDAs [16–19, 25]. This type of method uses the graph neural network model to learn embeddings for circRNA and disease entities, and then the embeddings of diseases and circRNAs are used to calculate the possibility of the association between them. For example, Wang et al. [16] proposed a method based on graph convolution network (GCN) for CDAs prediction (GCNCDA). Particularly, they used GCNCDA to predict the possible circRNAs related to breast cancer, glioma and colorectal cancer. Similarly, Bian et al. [17] proposed a method based on graph transformer network for CDAs prediction. However, most of the GNN-based methods [18, 19, 26] for CDAs prediction adopted homogeneous graph models, which regard the disease entities and circRNA entities as the same type of entity. Most of circRNAs regulate diseases by acting as sponge of microRNA (miRNA), a small number of circRNAs participate in the regulation of diseases by directly regulating genes. For example, Hsa_circ_0000285 [27] could contribute to gastric cancer progression by sponging miR-1278. On the contrary, CircGSK3B can inhibit the progression of gastric cancer by directly interacting with EZH2. Unfortunately, all previous CDAs prediction methods ignore the heterogeneity of different biomedical pathways, hence losing the ability to capture the underlying heterogeneous information. To capture such heterogeneity between different types of entities, we propose a novel graph neural network which is enhanced by our designed metapath based method. In particular, a metapath *P* (described in “Metapaths based on biomedical pathways” section) is defined by a sequence of entities between two types of entities, which can describe a composite relation between them.

In this work, we propose a heterogeneous graph neural network based on metapath for CDAs prediction (HMCDA). First, we construct a heterogeneous graph containing three types of entities (i.e circRNA, disease and miRNA). Afterwards, six metapaths based on biomedical pathways are defined to learn the embeddings of circRNA entities and disease entities. Finally, the embeddings of disease and circRNA are used to predict novel CDAs.

**Fig. 1:**
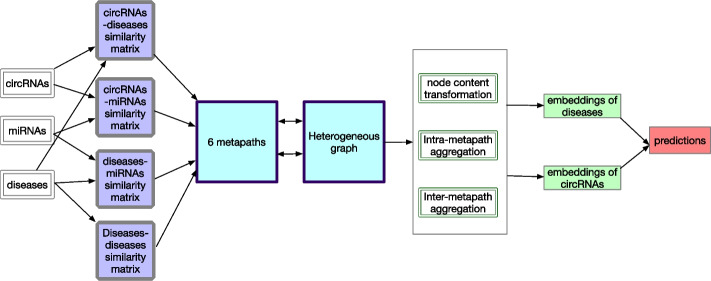
An overview of our proposed HMCDA model

## Methods

Figure 1 is an overview of our proposed HMCDA model. First, in “Construction of heterogeneous graph”   section, we construct a heterogeneous graph by integrating circRNA-disease associations (CDAs), circRNA-miRNA associations, disease-miRNA associations and disease-disease associations (DDAs). Besides, six metapaths based on biomedical pathways are defined in “Metapaths based on biomedical pathways” section  among circRNA, miRNA and disease entities. Afterwards, a metapath aggregated graph neural network is used to learn the embeddings of circRNA and disease entities through the node content transformation (“Node content transformation” section), intra-metapath aggregation (“Intra-metapath aggregation” section) and inter-metapath aggregation (“Inter-metapath aggregation” section). Finally, in “circRNA-disease associations prediction” section, the embeddings of circRNA and disease entities are used to predict the novel CDAs. We use Fig. 1 to illustrate the workflow of our proposed model.

### Construction of heterogeneous graph

2160 CDAs, 1964 circRNA-miRNA associations and 1964 disease-miRNA associations are obtained from CircR2Disease v2.0 [28]. Besides, 74 disease-disease associations are obtained form DisGeNET database [29] (Table 1). It should be noted that the DDAs in the DisGeNET database is calculated based on shared genes by followling formula:

1 $$\begin{aligned} \begin{aligned} {\text {Jaccard}}_{G}=\frac{G_{1} \cap G_{2}}{G_{1} \cup G_{2}}, \end{aligned} \end{aligned}$$

where G1 is the set of genes associated to disease 1, G2 is the set of genes associated to disease 2. As shown in Table 1, We construct a dataset consisting of the pairwise relationships between circRNAs, miRNAs and diseases. The dataset could be found in supplementary material 1. As shown in supplementary material 1, the table contains three types of entities (i.e. circRNA, disease and miRNA) and four types of associations (i.e. circRNA-disease association, circRNA-miRNA association, disease-miRNA association and disease-disease association). Each entity has its own ID. Based on this dataset, we construct a heterogeneous graph for the subsequent model training.

**Table 1:** Statistics of the dataset

Entity types	Num	Edge types	Num
circRNA	1556	circRNA-disease	2160
miRNA	840	circRNA-miRNA	1964
Disease	243	Disease-miRNA	1964
		Disease-disease	74
Total	2639		6162

### Metapaths based on biomedical pathways

A metapath P is defined by a sequence of entities between two types of entities, which can describe a composite relation between them. We define six types of metapaths according to biomedical pathways in this section.

$P_{cmc}$ (circRNA-miRNA-circRNA): two circRNAs are associated with the same miRNA by acting as miRNA sponge.

$P_{cmdmc}$ (circRNA-miRNA-disease-miRNA-circRNA): two circRNAs are associated with the same disease by acting as miRNA sponge.

$P_{cdc}$ (circRNA-disease-circRNA): two circRNAs are associated with the same disease by not acting as miRNA sponge.

$P_{dmd}$ (disease-miRNA-disease): two diseases are associated with the same miRNA.

$P_{dcd}$ (disease-circRNA-disease): one circRNA associated with two diseases through the circRNA-gene-disease pathway.

$P_{dd}$ (disease-disease): two diseases are associated with by sharing the same gene as calculated by Eq. (1).

### Node content transformation

**Fig. 2:**
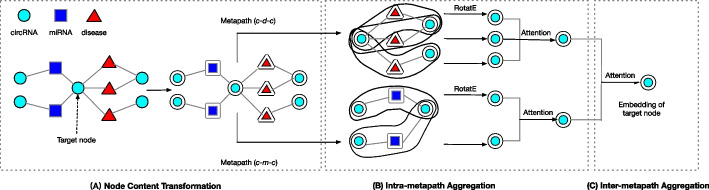
Flowchart of learning the embedding for target node. **A** Different types of entities were transformed into the same latent factor space by node content transformation. **B** All information in the same metapath with a same target entity is aggregated into target entity. **C** Information in different metapaths is aggregated into target entity

The feature vectors’ dimensions of different types of entities are different. As shown in Fig. 2A,in order to make the subsequent operation more efficient, we first use the following formula to transform the feature vector of different types of entities into the same latent space.

2 $$\begin{aligned} \begin{aligned} {\textbf{h}}_{v}^{\prime }={\textbf{W}}_{A} \cdot {\textbf{x}}_{v}^{A}, \end{aligned} \end{aligned}$$

where ${\textbf{x}}_{v} \in {\mathbb {R}}^{d_{A}}$ is the original feature vector of the entity *v*. A is the type of node (i.e. circRNA, miRNA and disease), ${d_{A}}$ is the number of nodes of type A and ${\mathbb {R}}^{d_{A}}$ is the dimension of node A. ${\textbf{h}}_{v}^{\prime }$ is the space vector after transpose of entity *v*. ${\textbf{W}}_{A}$ is the parametric weight matrix of type A’s entities. After entity content transformation, different types entities share the same latent factor space.

### Intra-metapath aggregation

We have defined six types of metapaths in “Metapaths based on biomedical pathways” section, and there are many metapath instances (e.g circRNA9119-miR26a-circ_0005105 [30, 31] is a metapath instance of metapath $P_{cmc}$) for each type of metapath. As shown in Fig. 2B, given a metapath P and target entity *v*, intra-metapath aggregation serves to aggregate all metapath instance information based on metapath P and target entity *v*. We denote a metapath instance by $\textrm{P}(v, u)$, where *v* is the target entity and $u \in {\mathcal {N}}_{v}^{\textrm{P}}$ is the metapath-based neighbor of the target entity *v*. To encode the information of metapath instance $\textrm{P}(v, u)$, we use a relational rotation encoder (RotatE) [32]. RotatE is a method for knowledge graph embedding proposed by Sun et al. In particular, RotatE can aggregate both the information of each entity in the metapath instance, and the order information of the entities. Given $\textrm{P}(v, u)=\left( t_{0}, t_{1}, \ldots , t_{n}\right)$ with $t_{0}=u$ and $t_{n}=v$, let $R_{i}$ be the relation between entity $t_{i-1}$ and entity $t_{i}$. Let ${\textbf{r}}_{i}$ be the relation vector of $R_{i}$, the relational rotation encoder is formulated as:

3 $$\begin{aligned}&{\textbf{o}}_{0} ={\textbf{h}}_{t_{0}}^{\prime }={\textbf{h}}_{u}^{\prime }, \\&{\textbf{o}}_{i} ={\textbf{h}}_{t_{i}}^{\prime }+{\textbf{o}}_{i-1}\odot {\textbf{r}}_{i}, \\&{\textbf{h}}_{P(v,u)} =\frac{{\textbf{o}}_{n}}{n+1},\\ \end{aligned}$$

where ${\textbf{h}}_{t_{i}}^{\prime }$ and ${\textbf{r}}_{i}$ are both complex vectors and $\odot$ is the element-wise product. For each metapath instance $P_i$, we obtain a single vector representation ${\textbf{h}}_{P_i(v, u)}$. Then, we adopt the attention mechanism [33] to calculate the weighted sum of the metapath instances of metapath $P_i$ related to target entity *v* as follows:

4 $$\begin{aligned} \begin{aligned} e_{v u}^{P_i}&={\text {LeakyReLU}}\left( {\textbf{a}}_{P_i}^{\top } \cdot \left[ {\textbf{h}}_{v}^{\prime } \Vert {\textbf{h}}_{P_i(v, u)}\right] \right) , \\ \alpha _{v u}^{P_i}&=\frac{\exp \left( e_{v u}^{P_i}\right) }{\sum _{s \in {\mathcal {N}}_{v}^{P_i}} \exp \left( e_{v s}^{P_i}\right) }, \\ {\textbf{h}}_{v}^{P_i}&=\sigma \left( \sum _{u \in {\mathcal {N}}_{v}^{P_i}} \alpha _{v u}^{P_i} \cdot {\textbf{h}}_{P_i(v, u)}\right) , \end{aligned} \end{aligned}$$

where ${\textbf{a}}_{\mathrm {P_i}} \in {\mathbb {R}}^{2 d^{\prime }}$ is the parameterised attention vector for each metapath instance, $e_{v u}^{P_i}$ and $\alpha _{v u}^{P_i}$ are the importances of metapath instance $P_i(v, u)$ to the target entity *v* and the corresponding normalized importance weight. Finally, the weighted sum and an activation function $\sigma (\cdot )$ are used to obtain the vector representation of node *v* based on metapath $P_i$ (i.e. ${\textbf{h}}_{v}^{P_i}$).

Particularly, we can also extend equation above by using the attention mechanism with *K* heads to prevent the overfitting problem.

5 $$\begin{aligned} \begin{aligned} {\textbf{h}}_{v}^{P_i}=\Vert _{k=1}^{K} \sigma \left( \sum _{u \in {\mathcal {N}}_{v}^{P_i}}\left[ \alpha _{v u}^{P_i}\right] _{k} \cdot {\textbf{h}}_{P_i(v, u)}\right) , \end{aligned} \end{aligned}$$

where $\left[ \alpha _{v u}^{P_i}\right] _{k}$ is the normalized importance in *k*th head.

Afterwards, we obtain a vector repression ${\textbf{h}}_{v}^{P_i}$ which aggregates the information of all metapath instances of *P* related to the target entity *v* through intra-metapath aggregation. In the next section, we will implement the inter-metapath aggregation to aggregate information in different metapaths into a target entity.

### Inter-metapath aggregation

In this section, we use the attention mechanism [as shown in Eq. (5)] again to aggregate information in different metapath into target entity. As shown in Fig. 2C we summarize each metapath $P_i$ with the same target entity type by the following formula:

6 $$\begin{aligned} \begin{aligned} \textrm{s}_{P_{i}}=\frac{1}{\left| {\mathcal {V}}_{A}\right| } \sum _{v \in {\mathcal {V}}_{A}} \tanh \left( {\textbf{M}}_{A} \cdot {\textbf{h}}_{v}^{P_{i}}+{\textbf{b}}_{A}\right) , \end{aligned} \end{aligned}$$

where ${\textbf{M}}_{A}$ and $\textrm{b}_{A}$ are learnable parameters.

After that, the attention mechanism is used to merge the information of different type metapaths as follows:

7 $$\begin{aligned} \begin{aligned} e_{P_{i}}&={\textbf{q}}_{A}^{\top } \cdot {\textbf{s}}_{P_{i}}, \\ \beta _{P_{i}}&=\frac{\exp \left( e_{P_{i}}\right) }{\sum _{P \in {\mathcal {P}}_{A}} \exp \left( e_{P}\right) }, \\ {\textbf{h}}_{v}^{{\mathcal {P}}_{A}}&=\sum _{P \in {\mathcal {P}}_{A}} \beta _{P} \cdot {\textbf{h}}_{v}^{P}, \end{aligned} \end{aligned}$$

where $\textrm{q}_{A} \in {\mathbb {R}}^{d_{A}}$ is the parameterised attention vector of type A’s entity. $e_{P_{i}}$ and $\beta _{P_{i}}$ are the importance of metapath $P_i$ to target entity and corresponding normalization importance. Then, the weighted sum is used to fuse the information of different metapath and obtain a vector repression ${\textbf{h}}_{v}^{{\mathcal {P}}_{A}}$. Finally, an additional linear transformation (i.e. ${\textbf{W}}_{o}$) and a nonlinear function (i.e. $\sigma (\cdot )$) are used to obtain the embedding of each entity:

8 $$\begin{aligned} \begin{aligned} {\textbf{h}}_{v}=\sigma \left( {\textbf{W}}_{o} \cdot {\textbf{h}}_{v}^{{\mathcal {P}}_{A}}\right) , \end{aligned} \end{aligned}$$

where ${\textbf{h}}_{v}$ is the embedding of a circRNA or disease entity.

### circRNA-disease associations prediction

Given embeddings of each disease entity (i.e. $h_{(d_i)}$) and each circRNA entity (i.e. $h_{(c_j)}$). We use the following formula to calculate the possibility (i.e. $p_{d_{i} c_{j}}$) that they link together:

9 $$\begin{aligned} \begin{aligned} p_{d_i c_j}=\sigma \left( {\textbf{h}}_{(d_i)}^{\top } \cdot {\textbf{h}}_{(c_j)}\right) \end{aligned} \end{aligned}$$

### Model training

To optimize our HMCDA model, we use the following loss function:

10 $$\begin{aligned} {\mathcal {L}}=-\sum _{(u, v) \in \Omega } \log \sigma \left( {\textbf{h}}_u^{\top } \cdot {\textbf{h}}_v\right) -\sum _{\left( u^{\prime }, v^{\prime }\right) \in \Omega ^{-}} \log \sigma \left( -{\textbf{h}}_{u^{\prime }}^{\top } \cdot {\textbf{h}}_{v^{\prime }}\right) , \end{aligned}$$

where $\Omega$ and $\Omega ^{-}$ are sets of positive and negative pairs.

## Results

In this section, we first present our experiment setup, where we detail our data split and the used evaluation metrics. Then, we present the result of the extensive experiment, ablation experiment and case study.

### Experiment setup

In this paper, the fivefold cross validation method is used to evaluate the performance of the model. All CDAs are divided into five subsets of equal size, with each subset selected in turn for testing and other four subsets for training. The testing set is used to test the generalization ability of all models and derive the receiver operating characteristic curve (ROC) and Precision–Recall (PR) curve. We also obtain the average area under the ROC (AUC) and the average area under PR curve (AUPR).

In addition, to train our model and all other baselines, we use the Pytorch package. In particular, we use the Adam optimizer to optimize all models. For the hyperparameters, we tune the learning rate in  $\left\{ 10^{-2},10^{-3},10^{-4} \right\}$; the latent dimension in $\left\{ 32,64,128 \right\}$ and the $L_{2}$ normalisation in $\left\{ 10^{-2}, 10^{-3}, 10^{-4}, 10^{-5} \right\}$. We define the negative samples as those nodes that are not linked together. In the training set, we randomly sample 5 different negative node pairs for each positive node pair.

### Extensive experiment

To demonstrate the performance of HMCDA, we choose four state of the art model to make an extensive comparison.

- *GATCL2CD* GATCL2CD [34] is a method based on heterogeneous graph attention network for CDAs prediction by fusing disease semantic similarity information, circRNA sequence similarity and function similarity.
- *iCircDA-MF* iCircDA-MF [35] is a CDAs prediction method based on matrix factorization by integrating information from circRNA similarity, disease semantic similarity and known CDAs.
- *GCNCDA* [36] GCNCDA is a GCN-based method for CDAs prediction by fusing disease semantic similarity information, disease and circRNA Gaussian Interaction Profile similarity.
- *GATNNCDA* [37] GATNNCDA is a method based on graph attention network and multi-layer neural network for CDAs prediction. Similar to GCNCDA [36], it also uses the disease semantic similarity information, disease and circRNA Gaussian Interaction Profile similarity.

As shown in Tables 2 and 3, the mean AUC values of HMCDA, iCircDA-MF, GCNCDA, and GATNNCDA are 0.9135, 0.8134, 0.7334, and 0.8234 respectively. HMCDA achieves the best AUC value 0.9135, which increases by 9.01% over the second-best method (i.e. GATNNCDA). Meanwhile, the mean AUPR values of HMCDA, iCircDA-MF, GCNCDA, and GATNNCDA are 0.9212, 0.8200, 0.7220 and 0.8317 respectively. Similarly, HMCDA achieves the best AUPR value of 0.9212, which increases by 8.95% over the sub-optimal method (i.e. GATNNCDA). Therefore, we can conclude that HMCDA can outperform competitive baselines and achieve state-of-the-art performance.

**Table 2:** AUCs of HMCDA under fivefold cross validation compared with four previous models

	HMCDA	GATCL2CD	iCircDA-MF	GCNCDA	GATNNCDA
Fold 1	0.8913	0.8322	0.8111	0.7307	0.8222
Fold 2	0.9096	0.8419	0.8095	0.7347	0.8191
Fold 3	0.9474	0.8404	0.8005	0.7389	0.8107
Fold 4	0.8977	0.8457	0.8199	0.7392	0.8270
Fold 5	0.9217	0.8104	0.8262	0.7235	0.8383
Mean	0.9135	0.83412	0.8134	0.7334	0.8234

**Table 3:** AUPRs of HMCDA under fivefold cross validation compared with four previous models

	HMCDA	GATCL2CD	iCircDA-MF	GCNCDA	GATNNCDA
Fold 1	0.9054	0.8431	0.8216	0.6999	0.8321
Fold 2	0.9214	0.8189	0.8200	0.7287	0.8240
Fold 3	0.9502	0.8302	0.8120	0.7398	0.8258
Fold 4	0.9046	0.8334	0.8205	0.7301	0.8377
Fold 5	0.9246	0.8024	0.8261	0.7119	0.8387
Mean	0.9212	0.8256	0.8200	0.7220	0.8317

### Ablation experiment

**Table 4:** Different combination of metapaths

	c–d–c	d–c–d	c–m–c	d–m–d	d–d	c–m–d–m–c
metapath2	✓	✓				
metapath3	✓	✓	✓			
metapath4	✓	✓	✓	✓		
metapath5	✓	✓	✓	✓	✓	
metapath6	✓	✓	✓	✓	✓	✓

Metapath2: Combination of 2 metapaths: $c-d-c, d-c-d$. Metapath3: Combination of 3 metapaths: $c-d-c, d-c-d, c-m-c$, Metapath4: Combination of 4 metapaths: $c-d-c, d-c-d, c-m-c, d-m-d$. Metapath5: Combination of 5 metapaths: $c-d-c, d-c-d, c-m-c, d-m-d, d-d$. Metapath6: Combination of 6 metapaths: $c-d-c, d-c-d, c-m-c, d-m-d, d-d, c-m-d-m-c$

**Fig. 3:**
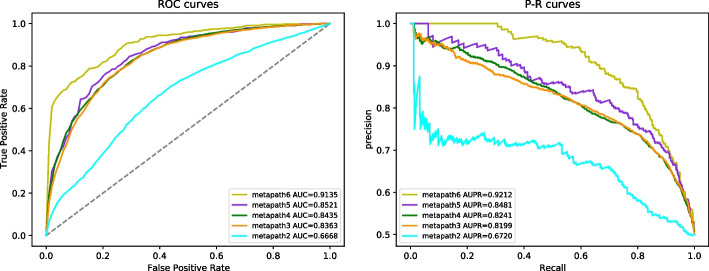
Result of ablation experiment

To evaluate the effectiveness of different biomedical pathways, we conduct the ablation experiment. As shown in Table 4, we construct five different combinations of metapaths and name them as metapath2, metapath3, metapath4, metapath5, and metapath6 according to the metapaths they contain.

As shown in Fig. 3, the performance of HMCDA improves with the increase of the number of metapaths. Besides, in addition to $c-d-c$ and $d-c-d$, $c-m-c$ should be the most important metapath. Compared with metapath2, the AUC and AUPR values of metapath3 have increased by 16.95% and 14.79% respectively. Similarly, $c-m-d-m-c$ should be the second important metapath. Compared with metapath5, the AUC and AUPR values of metapath6 have increased by 6.14% and 7.31% respectively. Two metapaths ($d-m-d$ and $d-d$) with disease as target nodes contribute less to the performance of the model compared with those with circRNAs as their target nodes. The cause of this observation may be that the similarities between the disease are more difficult to be learnt than the similarities between circRNAs.

### Case study

**Table 5:** The top 10 gastric cancer-related candidate circRNAs

Rank	circRNA name	CircR2Disease	PMID
1	circRNA_103516	None	None
2	hsa_circ_0044226	None	None
3	hsa_circ_0000285	None	PMC9169205
4	hsa_circ_001436	None	None
5	hsa_circ_0061140	None	None
6	circ_0001105	None	None
7	hsa_circ_0000615	None	PMC8161999
8	hsa_circ_0070616	None	None
9	circCSNK1G1	None	PMC8253995
10	Circ-03955	None	None

To test the utility of HMCDA, we first implement a case study on gastric cancer to present the top 10 predicted related circRNAs of gastric cancer. Gastric cancer is the fifth most common cancer and the third most common cause of cancer death globally [38]. Therefore, it is critical to find biomarkers and therapeutic targets related to gastric cancer. As shown in Table 5, CircR2Disease indicates whether the predicted association is already present in the CircR2Disease dataset. PMID is the evidence of the predicted association. Among the top 10 predicted gastric cancer-related circRNAs, three are not found in the data used for training, but verified by external articles (i.e. hsa_circ_0000285 [39], hsa_circ_0000615 [40], circCSNK1G1 [41]). Wang et al. [39] found that hsa_circ_0000285 contributes to gastric cancer progression by sponging miR-1278 and upregulating FN1. Dong et al. [40] found that the expression of hsa_circ_0000615 is decreased in gastric cancer tissue. In addition, Qiang et al. [41] found that CircCSNK1G1 can contribute to the tumorigenesis of gastric tancer by sponging miR-758 and Regulating ZNF217 expression. These results indicate that HMCDA has the ability to predict potential gastric cancer-related circRNAs.

**Table 6:** The top 10 hepatocellular carcinoma-related candidate circRNAs

Rank	circRNA name	CircR2Disease	PMID
1	circRNA-MTO1	None	None
2	circRNA_103516	None	None
3	hsa_circ_0005273	None	None
4	hsa_circ_0070269	Exist	31,606,623
5	circ_0062491	None	None
6	circ-Foxo3	None	None
7	circZNF652	None	31000195
8	circSDHC	None	None
9	circ_0008928	None	34220494
10	circRNA-51217	None	None

To test the utility of HMCDA in other diseases, we also implement a case study on hepatocellular carcinoma (HCC) and lung cancer. As shown in Table 6, among the top 10 predicted hepatocellular carcinoma-related circRNAs, two are not found in the data used for training, but verified by external researchs (i.e. circZNF652 [42] and circ 0008928 [43]). Guo et al. [42] foud that circZNF652 promotes hepatocellular carcinoma metastasis through inducing snail-mediated epithelial-mesenchymal transition by sponging miR-203/miR-502–5p. Besides, Wang et al. [43] found that circ_0008928 may be related to the synergistic anti-hepatocellular carcinoma effect of Berberine and regorafenib.

**Table 7:** The top 10 lung cancer-related candidate circRNAs

Rank	circRNA name	CircR2Disease	PMID
1	hsa_circ_0006772	None	None
2	CircHivep2	None	None
3	circABCB10	None	32,572,881
4	hsa_circ_0000267	Exist	None
5	circSIPA1L1	None	None
6	hsa_circRNA_101128	None	None
7	hsa_circ_0032463	None	None
8	hsa_circ_0000677	None	None
9	hsa_circ_0000467	None	None
10	hsa_circ_0002018	None	32,368,305

As shown in Table 7, among the top 10 predicted lung cancer-related circRNAs, two are verified by external researchs (i.e. circABCB10 [44] and has_circ_0002018 [45]). Hu et al. [44] found that circABCB10 could promote the proliferation and migration of lung cancer cells through down-regulating microRNA-217 expression. Besides, Xu et al. [45] found that has_circ_0002018 could supress the lung metastasis of breast cancer by sponging miR-658. These results indicate that HMCDA has the ability to predict potential related circRNAs in other diseases.

## Conclusions

In this work, we proposed a novel heterogeneous graph neural network which is enhanced by our designed six metapaths. We term our model as HMCDA and we use HMCDA to effectively predict the unseen associations between circNAs and diseases. Our extensive experiments in fivefold cross validation have indicated that our proposed HMCDA model can outperform four state-of-the-art circRNA-disease prediction models. In addition, our detailed case study suggests that HMCDA can effectively identify the novel CDAs.

### Supplementary Information

**Additional file 1**.

## Data Availability

The dataset and source code can be freely downloaded from: https://github.com/shiyangl/HMCDA.

## References

[1] Diener T (1971). Potato spindle tuber “virus”: IV. A replicating, low molecular weight RNA. Virology.

[2] Hsu M-T, Coca-Prados M (1979). Electron microscopic evidence for the circular form of RNA in the cytoplasm of eukaryotic cells. Nature.

[3] Zheng Y, Ji P, Chen S, Hou L, Zhao F (2019). Reconstruction of full-length circular RNAs enables isoform-level quantification. Genome Med.

[4] Dong R, Ma X-K, Li G-W, Yang L (2018). Circpedia v2: an updated database for comprehensive circular RNA annotation and expression comparison. Genom Proteom Bioinform.

[5] Zang X, Jiang J, Gu J, Chen Y, Wang M, Zhang Y, Fu M, Shi H, Cai H, Qian H (2022). Circular RNA EIF4G3 suppresses gastric cancer progression through inhibition of *β*-catenin by promoting *δ*-catenin ubiquitin degradation and upregulating SIK1. Mol Cancer.

[6] Yang G, Zhang Y, Lin H, Liu J, Huang S, Zhong W, Peng C, Du L (2022). CircRNA circ\_0023984 promotes the progression of esophageal squamous cell carcinoma via regulating miR-134-5p/cystatin-s axis. Bioengineered.

[7] Li T, Shao Y, Fu L, Xie Y, Zhu L, Sun W, Yu R, Xiao B, Guo J (2018). Plasma circular RNA profiling of patients with gastric cancer and their droplet digital RT-PCR detection. J Mol Med.

[8] Song J, Zheng J, Liu X, Dong W, Yang C, Wang D, Ruan X, Zhao Y, Liu L, Wang P (2022). A novel protein encoded by ZCRB1-induced circHEATR5B suppresses aerobic glycolysis of GBM through phosphorylation of JMJD5. J Exp Clin Cancer Res.

[9] Ledford H (2013). Circular RNAs throw genetics for a loop. Nat..

[10] Yang S, Zhou H, Liu M, Jaijyan D, Cruz-Cosme R, Ramasamy S, Subbian S, Liu D, Xu J, Niu X (2022). SARS-CoV-2, SARS-CoV, and MERS-CoV encode circular RNAs of spliceosome-independent origin. J Med Virol.

[11] Fang P, Jiang Q, Liu S, Gu J, Hu K, Wang Z (2022). Circ\_0002099 is a novel molecular therapeutic target for bladder cancer. Drug Dev Res.

[12] Luo R (2022). CircRNA circ-MYBL2 absorbs precursor miR-92b in the nucleus to suppress its role in enhancing gastric cancer cell proliferation. Am J Med Sci.

[13] Jeck WR, Sorrentino JA, Wang K, Slevin MK, Burd CE, Liu J, Marzluff WF, Sharpless NE (2013). Circular RNAs are abundant, conserved, and associated with ALU repeats. RNA.

[14] Barracchia EP, Pio G, D’Elia D, Ceci M (2020). Prediction of new associations between ncRNAs and diseases exploiting multi-type hierarchical clustering. BMC Bioinform.

[15] Wang L, You Z-H, Li J-Q, Huang Y-A (2020). IMS-CDA: prediction of CircRNA-disease associations from the integration of multisource similarity information with deep stacked autoencoder model. IEEE Trans Cybern.

[16] Wang L, You Z-H, Li Y-M, Zheng K, Huang Y-A (2020). GCNCDA: a new method for predicting circRNA-disease associations based on graph convolutional network algorithm. PLoS Comput Biol.

[17] Bian C, Lei X-J, Wu F-X (2021). GATCDA: predicting circRNA-disease associations based on graph attention network. Cancers.

[18] Wang L, You Z-H, Li Y-M, Zheng K, Huang Y-A (2020). GCNCDA: a new method for predicting circRNA-disease associations based on graph convolutional network algorithm. PLoS Comput Biol.

[19] Bian C, Lei X-J, Wu F-X (2021). GATCDA: predicting circRNA-disease associations based on graph attention network. Cancers.

[20] Liu S, Meng Z, Macdonald C, Ounis I (2023). Graph neural pre-training for recommendation with side information. ACM Trans Inf Syst.

[21] Liu S, Ounis I, Macdonald C, Meng Z. A heterogeneous graph neural model for cold-start recommendation. In: Proceedings of the 43rd international ACM SIGIR conference on research and development in information retrieval. 2020;2029–2032.

[22] Yin N, Shen L, Wang M, Luo X, Luo Z, Tao D. Omg: towards effective graph classification against label noise. IEEE Trans Knowl Data Eng. 2023.

[23] Yi Z, Ounis I, Macdonald C. Graph contrastive learning with positional representation for recommendation. In: European conference on information retrieval. Springer; 2023. p. 288–303.

[24] Li M, Liu M, Bin Y, Xia J (2020). Prediction of circRNA-disease associations based on inductive matrix completion. BMC Med Genom.

[25] Liu S, Ounis I, Macdonald C. An mlp-based algorithm for efficient contrastive graph recommendations. In: Proceedings of the 45th international ACM SIGIR conference on research and development in information retrieval; 2022. p. 2431–2436.

[26] Liu S. Effective graph representation learning for ranking-based recommendation. Ph.D. thesis, University of Glasgow; 2023.

[27] Wang X, Tan M, Huang H, Zou Y, Wang M (2022). Hsa\_circ\_0000285 contributes to gastric cancer progression by upregulating FN1 through the inhibition of miR-1278. J Clin Lab Anal.

[28] Fan C, Lei X, Tie J, Zhang Y, Wu F, Pan Y (2021). Circr2disease v2.0: an updated web server for experimentally validated circRNA-disease associations and its application. Genom Proteomics Bioinform.

[29] Piñero J, Bravo À (2017). Queralt-Rosinach N, Gutiérrez-Sacristán A, Deu-pons J, Centeno E, García-Grcía J, Sanz F, Furlong LI. DisGeNET: a comprehensive platform integrating information on human disease-associated genes and variants. Nucleic Acids Res.

[30] Zhang L, Liu X, Che S, Cui J, Liu Y, An X, Cao B, Song Y (2018). CircRNA-9119 regulates the expression of prostaglandin-endoperoxide synthase 2 (PTGS2) by sponging miR-26a in the endometrial epithelial cells of dairy goat. Reprod Fertil Dev.

[31] Wu Y, Zhang Y, Zhang Y, Wang J-J (2017). CircRNA hsa\_circ\_0005105 upregulates NAMPT expression and promotes chondrocyte extracellular matrix degradation by sponging miR-26a. Cell Biol Int.

[32] Sun Z, Deng Z-H, Nie J-Y, Tang J. Rotate: knowledge graph embedding by relational rotation in complex space; 2019. arXiv:1902.10197

[33] Vaswani A, Shazeer N, Parmar N, Uszkoreit J, Jones L, Gomez AN, Kaiser Ł, Polosukhin I. Attention is all you need. Adv Neural Inf Process Sys. 2017;30.

[34] Peng L, Yang C, Chen Y, Liu W (2023). Predicting circRNA-disease associations via feature convolution learning with heterogeneous graph attention network. IEEE J Biomed Health Inform.

[35] Wei H, Liu B (2020). iCircDA-MF: identification of circRNA-disease associations based on matrix factorization. Brief Bioinform.

[36] Wang L, You Z-H, Li Y-M, Zheng K, Huang Y-A (2020). GCNCDA: a new method for predicting circRNA-disease associations based on graph convolutional network algorithm. PLoS Comput Biol.

[37] Ji C, Liu Z, Wang Y, Ni J, Zheng C (2021). GATNNCDA: a method based on graph attention network and multi-layer neural network for predicting circRNA-disease associations. Int J Mol Sci.

[38] Smyth EC, Nilsson M, Grabsch HI, van Grieken NC, Lordick F (2020). Gastric cancer. Lancet.

[39] Wang X, Tan M, Huang H, Zou Y, Wang M (2022). Hsa\_circ\_0000285 contributes to gastric cancer progression by upregulating FN1 through the inhibition of miR-1278. J Clin Lab Anal.

[40] Dong Z, Liu Z, Liang M, Pan J, Lin M, Lin H, Luo Y, Zhou X, Yao W (2021). Identification of circRNA-miRNA-mRNA networks contributes to explore underlying pathogenesis and therapy strategy of gastric cancer. J Transl Med.

[41] Qiang F, Li J (2021). CircCSNK1G1 contributes to the tumorigenesis of gastric cancer by sponging miR-758 and regulating ZNF217 expression. Cancer Manag Res.

[42] Guo J, Duan H, Li Y, Yang L, Yuan L (2019). A novel circular RNA circ-ZNF652 promotes hepatocellular carcinoma metastasis through inducing snail-mediated epithelial-mesenchymal transition by sponging miR-203/miR-502-5p. Biochem Biophys Res Commun.

[43] Wang K, Yu G, Lin J, Wang Z, Lu Q, Gu C, Yang T, Liu S, Yang H (2021). Berberine sensitizes human hepatoma cells to regorafenib via modulating expression of circular RNAs. Front Pharmacol.

[44] Hu T, Zhu Q, Duan Q, Jin X, Wu R (2020). CircABCB10 promotes the proliferation and migration of lung cancer cells through down-regulating microRNA-217 expression. Eur Rev Med Pharmacol Sci.

[45] Xu G, Ye D, Zhao Q, He R, Ma W, Li Y, Tang S, Zhou Z, Li X, Zhang Z (2020). circNFIC suppresses breast cancer progression by sponging miR-658. J Cancer.

